# Responsive Cells for rhEGF bioassay Obtained through Screening of a CRISPR/Cas9 Library

**DOI:** 10.1038/s41598-019-40381-4

**Published:** 2019-03-07

**Authors:** Xi Qin, Wenrong Yao, Xinchang Shi, Lan Liu, Fang Huang, Youxue Ding, Yong Zhou, Lei Yu, Chuncui Jia, Shanhu Li, Chunming Rao, Junzhi Wang

**Affiliations:** 1 Department of Recombinant Products, National Institutes for Food and Drug Control, Beijing, 100050 China; 2 Department of Cell Engineering, Beijing Institute of Biotechnology, Beijing, 100850 China

## Abstract

Bioassay of recombinant protein products is important tests to ensure protein effectiveness. Some recombinant protein products have no cells used in their bioassay but instead use animal models, while others have no suitable method. Here, we developed a method to obtain responsive cells used in bioassay of proteins. After screening of a CRISPR/Cas9 library, we obtained a responsive cell line that grew faster in the presence of rhEGF (recombinant human epidermal growth factor) than that of control cells. We used this cell line for bioassay of rhEGF. This cell line, compared with the control cells, had a 2 day shorter operation time and had lower interference. The responsive cell line is more suitable for use in bioassay of rhEGF.

## Introduction

In 1962, a specific peptide, later termed epidermal growth factor (EGF), was discovered that could promote eyelid opening and teething of neonatal mice^1,2^. EGF is a 53 amino peptide with a molecular weight of approximately 6.05 kD^3,4^. EGF is a member of the growth factor family and can promote cell division and is thereby closely associated with some cancers. This peptide also plays an important role in respiratory and reproductive systems, can accelerate the process of wound healing^5^, and promote the growth of various epidermal tissues. Given this, EGF is widely used in clinical treatments and cosmetology. Recombinant human epidermal growth factor (rhEGF) can be obtained through gene engineering to transfer the human epidermal growth factor gene into yeast cells. RhEGF was approved by China Food and Drug Administration for external use and for eye use to treat burns, ulcers, as well as other traumas and corneal injuries.

Accurate determination of the potency of therapeutic rhEGF is crucial to ensure the safety and efficacy of the drug. A NIH3T3 cell proliferation assay is routinely used for bioassay of rhEGF. However, NIH3T3 cells are semi-dependent on rhEGF, resulting in poor response, high onset concentrations needed, a large variability of results, and a long experimental period (e.g., 6 days).

Transgene cell lines have widely been used as a more simple, reliable, and efficient method^6–9^ to determine a bioassay of recombinant products, as well as IFNα(Interferon α)^10^, BNP (Brain natriuretic peptide)^11^, EPO(Erythropoietin)^12^, and even some antibodies^13,14^. These methods have been developed based on their individual, extensively-studied signal pathways. In order to develop a method to obtain cells for bioassay of recombinant products with signal pathways that are still unknown, we screened cells with a CRISPR-Cas9 library given that this technique can be used as a powerful tool for high-throughput screening in genomes.

The system of RNA-guided CRISPR (clustered regularly interspaced short palindrome repeats)–associated nuclease Cas9 can induce double-stranded DNA breaks (DSBs) though the two specific activity sites of Cas9 (i.e., the sites of HNH and RuvC, which cut complementary and antisense strands, respectively) by the guiding of single guide RNA (sgRNA) to the specific target sequence in the genome. Then, DSBs initiate the process of DNA repair, which can either be based upon homologous recombination (HR) or non-homologous end jointing (NHEJ), causing DNA indel, repair, or replacement in the genome^15^. CRISPR-Cas9, also called the third generation of gene editing technology, functions as an adaptive immune system in bacteria^16^ but can also be used to introduce targeted loss-of-function mutations at specific sites in genomes^2,8,9,17,18^ and provides an effective means of screening mammalian cells with phenotypes of interest for multiple usages^19–24^. The Mouse CRISPR Knockout Pooled Library (GeCKO v2) is constructed with sgRNAs, Cas9, and puromycin in lentiviral vectors. The genome-wide GeCKO v2 mouse library target 20611 genes with 130209 unique guide sequences which could target to 5′ constitutive exons of genes in the mouse genome^25^.

Here, we conducted a CRISPR-based positive-selection screen to obtain a more responsive NIH3T3 cell line to rhEGF after introducing loss-of-function mutations via a CRISPR-Cas9 method. We packaged lentivirus packaging plasmids as a lentivirus library (LentiCRISPR) and infected NIH3T3 cells. After induction by low doses of rhEGF, clones that grew faster in the presence of rhEGF were screened as responsive cells to rhEGF. Using these responsive cells, we established a method for rhEGF bioassay, and we show that the new assay is reproducible, precise, and robust, thereby representing a viable alternative method to replace the traditional assay.

## Results

### Responsive cells and validation

**Figure 1:**
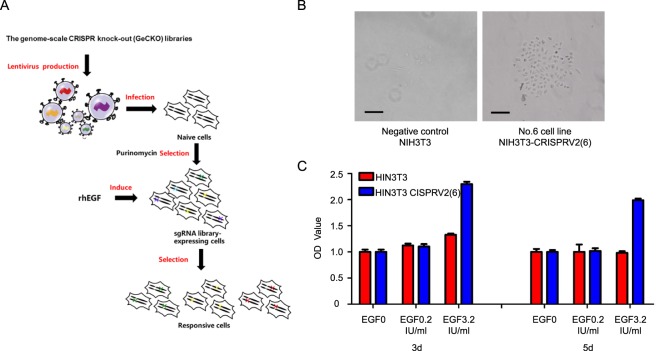
Screening of the responsive cell lines. (**A**) A schematic of forward genetic screens in NIH3T3 cells using pooled sgRNA libraries. (**B**) NIH3T3-CRISPRV2(6) growth faster than NIH3T3 with the presence of rhEGF (5 IU/mL). Scale bar, 60 mm. (**C**) NIH3T3-CRISPRV2(6) and NIH3T3 cells were treated with 0.2IU and 3.2IU EGF for 5 days, cell number was determined using the CCK8 assay. Statistical significance was determined using paired Student t test; *P < 0.05.

To obtain responsive cells for the rhEGF bioassay, we designed a CRISPR–Cas9-based positive-selection screen for regulation genes whose loss allowed cells to grow faster in the presence of 3.2 IU/mL rhEGF, which is a concentration of rhEGF that weakly increases cell proliferation, as illustrated in Fig. 1A.

We seeded cells infected with LentiCRISPR into 96-well plates (with one cell per well and seeded by flow cytometry). We then caused induction with rhEGF at a low concentration (3.2 IU/ml rhEGF). One clones that grew faster than negative controls in inducible media (Fig. 1B) were selected. CCK-8 was used to detect the clones, and we found that the growth of NIH3T3-CRISPRV2 (6) was not only 2 times faster than negative controls in inducible media but was also the same to the negative control in growth media (Fig. 1C). NIH3T3-CRISPRV2 (6) was more responsive to rhEGF than NIH3T3.

**Figure 2:**
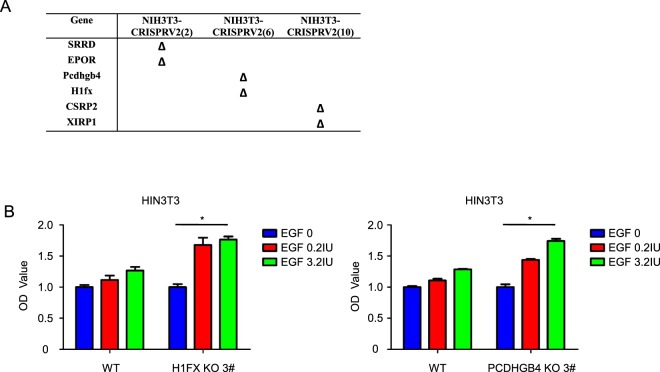
Validation of the responsive cell lines. (**A**) List of genes in which mutations were observed at target sites in the three colonies that grew faster than negative controls in inducible media. (**B**) Nontargeting (WT) control, H1FX-null and Pcdhgb4-null NIH3T3 cells were treated with 0.2IU and 3.2IU EGF for 5 days, cell number was determined using the CCK8 assay. Statistical significance was determined using paired Student t test; *P < 0.05.

We found, after comparing the results of second-generation sequencing (Fig. 2A), that genes changed the most were Pcdhgb4 and H1fx in NIH3T3-CRISPRV2 (6), and the sgRNAs were ATAGTCTGTGTTTCACTACC and GCGCCCTCGCTAGGGCCCGA. To validate whether these two genes could affect the response of NIH3T3 to rhEGF, we used their sgRNAs and Cas9 to inactivate the genes separately and used CCK-8 to detect the growth of these two mutants. Results shows that inactivation of the genes for Pcdhgb4 and H1fx could advance the response of NIH3T3 to rhEGF separately (Fig. 2B).

### Establishment and optimization of the new assay (CRI-3T3)

**Figure 3:**
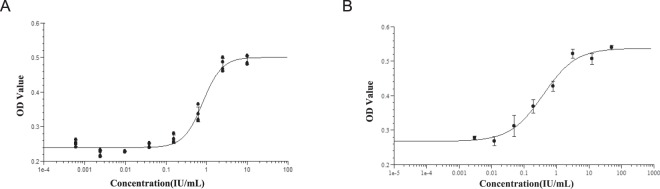
Establishment of the method used to detecting the bioassay of rhEGF with the responsive cell lines. (**A**) The new method to detect the bioassay of rhEGF using NIH3T3-CRISPRV2(6) cell lines (CRI- 3T3). NIH3T3-CRISPRV2(6) cells were treated with gradient concentrations of rhEGF. Each point and error bar represents the mean and standard deviation of three replicates, respectively. (**B**) The used method to detect the bioassay of rhEGF using NIH3T3 cell lines(3T3). NIH3T3 cells were treated with gradient concentrations of rhEGF. Each point and error bar represents the mean and standard deviation of three replicates, respectively.

To obtain stronger reactivity, stability, and better accuracy, crucial experimental parameters of the existing method (3T3) were subsequently optimized. Given that FBS contains various cytokines that could interfere with the effect of rhEGF on cells, the concentration of FBS in the assay media and the cells being starved via PBS prior to the reaction with rhEGF was necessary. Results showed that the PBS starvation and incubation in assay media (i.e., RPMI-1640 containing 0.2% FBS) without rhEGF for 24 h, along with the subsequent steps, was sufficient to maintain the survival of NIH3T3-CRISPRV2 (6) cells and a low-level of interfere on rhEGF function. Other parameters, including the number of NIH3T3-CRISPRV2 (6) cells, the rhEGF working concentration, and stimulation time were also optimized (Table 1). The EC_50_ (50% effective concentration) was 0.2959 IU/mL, and the signal-to-noise ratio was 1.845 under the optimized conditions (Fig. 3A). Compared with the method of 3T3 (Fig. 3B), the method of CRI-3T3 had a shorter performance time (i.e., 2 days), the concentration of FBS in the assay media was half of that in 3T3, and the initial concentration of rhEGF was 5 times lower than that of 3T3.

**Table 1:** Optimized Parameters for CRI-3T3.

Experimental Parameters	Optimal Values
Cell number (per well)	6000
Cell starvation time	10–30 m
Initial concentration of rhEGF	10 IU/mL
rhEGF dilution multiple	4
rhEGF stimulation time	48 h
EC_50_	0.2959 IU/mL

### Method validation of CRI-3T3

The National Standard of rhEGF was used as a reference for activity to estimate the potency of rhEGF within two separate batches. The National Standard and experimental rhEGF were each tested in triplicate for each dosage to validate CRI-3T3. All tests were conducted according to ICH (International Conference on Harmonization of Technical Requirements for Registration of Pharmaceuticals for Human Use) Guidelines and included accuracy, precision, specificity, stability of cell line, and the agreement between CRI-3T3 and 3T3 methods.

### Accuracy

Accuracy expresses the closeness of agreement between the measured results and the expected values. The accuracy was calculated as the percentage of rhEGF National Standard recovered from rhEGF formulations. For this, we used 2 batches of rhEGF and mixed this with 50% rhEGF National Standard separately to dilute the initial concentration. The ratio of the measured to the expected value was calculated after obtaining potency results with the methods of CRI-3T3 and 3T3. CRI-3T3 was repeated 7 times, while 3T3 was repeated 5 times. As shown in Table 2, the recovery rates of sample 1 were between 85.8–107.8% with the CRI-3T3 method and between 79.9–100.7% with the 3T3 method, while the coefficient of variation (CV) values were 11.4% and 11.5%, respectively. Recovery rates of sample 2 were between 87.2–103.0% with the CRI-3T3 method and between 88.5–104.7% with the 3T3 method, while the CV values were 8.3% and 8.4%, respectively. These results show that the new method (CRI-3T3) has higher accuracy and is suitable for routine rhEGF bioassay detection.

**Table 2:** Comparison of the two methods for recovery ratios.

	CRI-3T3 (n = 7)		3T3 (n = 5)	
	Sample 1	Sample 2	Sample 1	Sample 2
Mean	96.8	95.1	90.3	96.6
Std. Deviation	11.0	7.9	10.4	8.1
CV (%)	11.4	8.3	11.5	8.4

### Precision

In order to determine the repeatability of the new method (CRI-3T3), we performed the assay on 3 different days with 2 batches of rhEGF, and we repeated the analyses 3 times each so that we could better understand the plate-to-plate variability and inter-assay variation in these tests. As shown in Table 3, the intra-assay (i.e., within-day) CV of sample 1 was between 6.2–13.6%, and the inter-assay (i.e., between-day) variation was 12.0%. The intra-assay CV of sample 2 was between 1.0–4.0%, and the inter-assay variation was 6.3%. Runs on the different dates indicate that the CRI-3T3 method is repeatable.

**Table 3:** Repeatability of CRI-3T3 as used for rhEGF bioassay.

	Sample 1				Sample 2			
	Plate 1	Plate 2	Plate 3	Intra-assay CV (%)	Plate 1	Plate 2	Plate 3	Intra-assay CV (%)
Day 1	11085	10238	13284	13.6	14654	14924	14706	1.0
Day 2	15497	15094	15943	2.7	12909	13767	13139	3.4
Day 3	12790	13531	14475	6.2	15847	14741	15800	4.0
Mean	13124	12954	14567	—	14470	14477	14548	—
Inter-assay CV (%)	12.0%			—	6.3%			—

**Figure 4:**
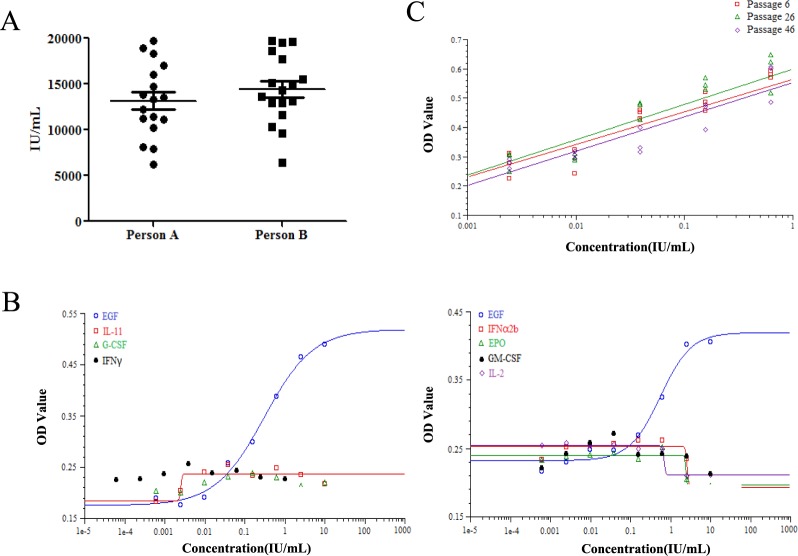
The validation of the new method CRI-3T3. (**A**) Agreement between the results performed by two persons analysed with *Mann-Whitney test* (n = 17, P = 0.3223). (**B**) Specificity of the assay. Responsiveness of NIH3T3-CRISPRV2(6) cells to a variety of different cytokines including rhEGF, rhIL-11, rhG-CSF, rhIFNg, rhIFNa2b, rhEPO, rhGM-CSF and rhIL-2 as well as aggregated rhEGF were detected. Each column illustrates the mean of three replicates. (**C**) Stability of NIH3T3-CRISPRV2(6) cell lines. The analyses of rhEGF samples were performed on cells at three different stages (passage #6, 26, 46) respectively. The graphs depict the linear range of dose-response curves.

Another analysis was carried out to determine the reproducibility of the CRI-3T3 method with two different personnel, and the assay was repeated 17 times separately. Data shows that there was no statistically significant difference between the effect when analyzed by multiple people (Fig. 4A) (p = 0.32).

### Specificity of the assay

Experiments were conducted to determine the specificity of the assay in the presence of other cytokines and their effect on the growth of NIH3T3-CRISPRV2 (6). Cytokines investigated included rhEGF, rhEPO, rhG-CSF, rhGM-CSF, rhIL-2, rhIL-11, rhIFN-γ, and rhIFN-α2b. As shown in Fig. 4B, none of the cytokines tested had a dose-effect relationship with NIH3T3-CRISPRV2 (6) except rhEGF.

### Stability of the NIH3T3-CRISPRV2 (6) cell line

The stability of the NIH3T3-CRISPRV2 (6) cell line and the response to EGF is crucial for the cells to be used in the bioassay of rhEGF. Stability was evaluated by comparing the responsiveness of cells at three different stages (at passage 6, 26, and 46). Results show that the cells behaved nearly indistinguishably at the different stages (Fig. 4C). The CV of the three potency estimates from the cells at different stages was 5.84%, illustrating the high stability of NIH3T3-CRISPRV2 (6) cells responding to rhEGF.

### Comparison of CRI-3T3 with 3T3

**Figure 5:**
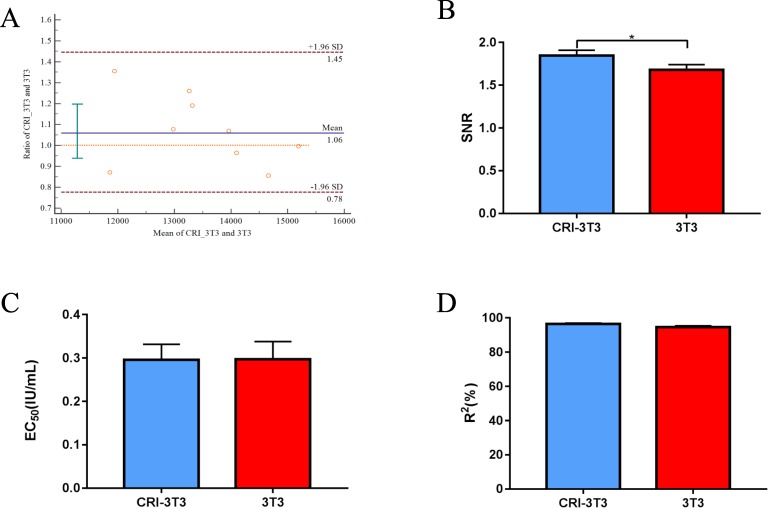
Compare the new method CRI-3T3 with 3T3. (**A**) Compared CRI-3T3 with 3T3 in detective values. Each plot represents the ratio of CRI-3T3 vs. 3T3 assay. The upper and lower dotted lines define the agreement limits within which 95% of differences between the two methods are expected to lie, and the middle dotted line represents the average ratio of CRI-3T3vs. 3T3assay (P = 0.3074). (**B**) Compared CRI-3T3 with 3T3 in signal-to-noise ratio. CRI-3T3 was performed 45 times and 3T3 was performed 27 times (P = 0.0165). (**C**) Compared CRI-3T3 with 3T3 in EC_50._ CRI-3T3 was performed 45 times and 3T3 was performed 27 times (P = 0.8097). (**D**) Compared CRI-3T3 with 3T3 in R^2^. CRI-3T3 was performed 45 times and 3T3 was performed 27 times (P = 0.0889).

A batch of rhEGF was tested by both the CRI-3T3 and 3T3 methods for multiple separate runs to compare the consistency of the two methods, quantification limit, linearity, and sensitivity. With the Bland-Altman analysis, the mean values measured from the two separate methods had no significant difference (p = 0.31) (Fig. 5A). The quantification limit (signal-to-noise ratio), linearity (R^2^), and sensitivity (EC_50_) were compared by repeating CRI-3T3 45 times and 3T3 27 times. The average signal-to-noise ratio was significantly different between CRI-3T3 and 3T3, with values of 1.85 and 1.66, respectively (p = 0.02) (Fig. 5B). The average EC_50_ was not significantly different between CRI-3T3 and 3T3, with values of 0.2959 and 0.2972, respectively (p = 0.81) (Fig. 5C). The average R^2^ also showed no significant difference between CRI-3T3 and 3T3, with values of 96.6% and 94.6%, respectively (p = 0.09) (Fig. 5D).

## Discussion

Usually, there are animal or cell methods for protein bioassay detection. Using cell methods to detect protein bioassay is a better choice, for the animal method had many problems, like animal welfare, complex, long cycle and large variability. But some protein products had no cell methods to detect their bioassays, or the cells had low reactivity to protein products, resulting in long cycle and large variability. So, transgene cell lines were used to detect protein bioassay. Using the transgene cell lines with reporter gene is the simple method to detect protein bioassay, but only the protein products which has clear and non-interfere signal pathways can use this method. So, we used the method of introducing loss-of-function mutations via a CRISPR-Cas9, and first used in construct responsive cell line to detect protein bioassay. This method could develop responsive cell lines to detect the bioassay of protein which not only had the clear signal pathway, but also had unknown or too complex signal pathways.

The compound rhEGF has been prescribed in Chinese pharmacopoeia for the treatment of burns, ulcers, various types of trauma, and corneal injuries. Accurate determination of the potency of therapeutic rhEGF is crucial for the safety and efficacy of the drug. The potency of this drug is currently determined by NIH3T3 cells with MTT staining, in which the inhibitory activity of rhEGF on acceleration of the proliferation of NIH3T3 cells is measured^26^. This method is known to be time-consuming, with high onset concentrations needed, and a large variability of results as rhEGF is semi-dependent on NIH3T3. Given this, the response of NIH3T3 to rhEGF needs to be improved.

CRISPR-Cas9 is a powerful tool to perform high-throughput screening for functional genomics^27^. This system can be packaged with lentivirus to infect cells with high efficiency^28^. The value of MOI (i.e., the number of viruses/number of cells) must be controlled to avoid too high or too low concentration of viruses to infect cells. After investigation, we determined the MOI value of 0.3 is suitable, as it neither led to too many lentiviruses infecting a cell to interfere with analysis or validation nor did it lead to too many non-infected cells.

In this study, a novel method was developed employing a NIH3T3-CRISPRV2 (6) cell line screened from NIH3T3 cells infected with LentiCRISPR which were induced by a low dose of rhEGF, and subsequently validated for precision, specificity, robustness, as well as agreement with the original assay. Our analyses resulted in both intra- and inter-assay variation less than 15.0%, high recovery rates, and no significant between-person effect, demonstrating suitable precision and accuracy that are better than the currently-used method. None of the eight cytokines used in our study, except rhEGF, had a dose-effect relationship with NIH3T3-CRISPRV2 (6), suggesting good specificity. Three stages of NIH3T3-CRISPRV2 (6) (passages 6, 26, and 46) showed no statistically significant difference in potency estimates and inter-passage CV, suggesting no effect of the passage number on responsiveness to rhEGF in this new assay. Bland–Altman analysis suggested that the results of new method (CRI-3T3) were highly consistent with the routine method (3T3). The optimized assay could be completed within 4 days, which is 2 days shorter than the routine analysis. The onset concentration of rhEGF is 20% of that needed in the routine analysis, and the concentration of fetal bovine serum in assay media is half of that needed in the routine analysis. These results suggest a high responsive to rhEGF in this assay and a reduction of the interference of supplementary materials in rhEGF products and cytokines in bovine serum. Though the second-generation sequencing and transcriptional heatmaps, we found the loss-of-function genes were Pcdhgb4 and H1fx in NIH3T3-CRISPRV2 (6), regardless of variation of EGF receptor. The EC_50_ showed no significant difference between the two methods, suggesting a similar sensitivity of the two cell lines used in our study, as well as no variation between EGF receptors. We surmised that the responsiveness promoted in NIH3T3-CRISPRV2 (6) is contributed to by changes of Pcdhgb4 or H1fx on the signal pathway of EGF, and NIH3T3 cells with loss-of-function of Pcdhgb4 and H1fx separately confirmed this.

## Conclusions

In conclusion, we obtained a NIH3T3 cell line with more responsiveness to rhEGF after introducing loss-of-function mutations via a CRISPR-Cas9 method. In addition, we established a method for rhEGF bioassay with suitable specificity, precision, accuracy, and robustness. The new assay showed a high consistency with the routine assay. This new method has many advantages over the routine assay, including a shorter operation time and lower interference. This method could be used as a routine analysis assay and employed in determination of rhEGF product potency. It could also be used in potency determination of other cytokines that share a receptor with EGF and in detection of neutralizing antibodies against therapeutic EGF in clinical serum samples. Other potential functions of Pcdhgb4 and H1fx need further research. The method of introducing loss-of-function mutations via a CRISPR-Cas9 could also to construct other responsive cell lines used in potency determination of their corresponding protein products.

## Methods

### Construction of LentiCRISPR

Mouse CRISPR Knockout Pooled Library (GeCKO v2) plasmid 100 ng was introduced into competent cells of Escherichia coli by electroporation. Selected with coating on ampicillin resistant LB plate, collected the ampicillin resistant clones and extracted the plasmid library. Co-transfected 120 ug plasmid library and lentivirus packaging plasmids to HEK293 cells (cultured with DMEM containing 10% FBS), collected the virus 24 h later and frozen in minus 70 °C as LentiCRISPR^29^. The titer of virus was determined: the total number of initial cells × the number of purinomycin resistant cells/(the number of cells before adding purinomycin × the volume of virus).

### CRISPR–based screens

The LentiCRISPR was infected into NIH3T3 cells at a MOI(multiplicity of infection) value of 2, incubated for 4 h, and cells were subsequently cultured in growth media (RPMI-1640 containing 10% FBS) for 48 h. Cells were selected in selective media (i.e., growth media containing 4 μg/mL purinomycin) for 1 week. Surviving cells were resuspended and seeded into 96-well plates with one cell per well by flow cytometry and incubated with growth media for 24 h. Media was replaced with inducible media (i.e., selective media containing 3.2 IU/mL rhEGF) for approximately 3–4 weeks. Selected clones grew faster than negative ones (i.e., NIH3T3 cells without infecting LentiCRISPR) when cultured in inducible media. Selected clones were amplified and validated using CCK-8. Responsive cells were selected that not only grew faster in inducible media but also grew at the same rate as negative cells in growth media. Genomes of responsive NIH3T3-CRISPRV2 (2), NIH3T3-CRISPRV2 (6), NIH3T3-CRISPRV2 (10) and NIH3T3 cells were extracted. After amplifying the sgRNA with two primer pairs, products were sent for second-generation sequencing.

### Generation of knockout cell lines

The sgRNA oligonucleotide pairs against Pcdhgb4 and H1fx (Pcdhgb4 5′-ACAATTTGCAGATTCAGCG G-3′, 5′-AGCTCAGATTGAGGGTCCGG-3′, 5′-CTTGTTTTGGAAG CAAAGGA-3′; H1fx 5′-CGCGAAGAAAGTGAAGAAGG-3′, 5′-TC TGGCGCGCATCTACGCTG-3′, 5′-TGGAGACCATCCGCAAGCTG- 3′) were phosphorylated, annealed, and cloned into LentiCRISPRv2-puro. Lentiviruses were produced in HEK293 cells by transfecting a 80% confluent 100 mm culture dish with 6 ug DNA (3ug Pcdhgb4/H1fx, 2.25 ug psPAX2, and 0.75 ug pMD2.G). DNA was mixed with 40 uL of lipofectamine 3000 in 300 uL Optimem media (Invitrogen, US). Media containing viruses was collected and filtered 48 h after transfection. NIH3T3 cells were kept in virus-containing media with Polybrene (6 μg/mL) for 24 h. Cells were allowed to recover in fresh media for 48 h before the media was replaced with the selection media containing puromycin (2 ug/mL). The cells were kept in selection media for 3 days before use in experiments, unless stated otherwise. Pcdhgb4 and H1fx knockouts in HIN3T3 cells were single-cell sorted into wells of a 96-well plate using a BD FACSAria cell sorter (BD Biosciences, US) to establish clonal knockout cell lines. Cells were grown for 2 weeks, and the resultant colonies were cultured.

### CCK-8 assay

Cells were seeded in 96-well plates at a density of 2000 cells per well and cultured in DMEM containing 10% FBS. Once attached, cells were starvation with the DMEM and cultured for 12 h. Cells were then treated with EGF (3.2 IU) and cultured for the indicated times. After indicated treatment(s), cell proliferation was determined via the Cell Counting Kit-8 (CCK-8) assay (Dojindo Laboratories, Tokyo, Japan) according to the manufacturer’s instructions. The absorbance of individual wells was determined at 450 nm. The OD value of the treatment group was normalized to values from the untreated control group. All reactions were performed in triplicate.

### NIH3T3-CRISPRV2 (6) based assay (CRI-3T3)

Plates (96-well Costar) were seeded with 6000 NIH3T3-CRISPRV2 (6) cells in assay media (RPMI-1640 containing 0.2% FBS) and incubated at 37 °C with 5% CO_2_ for 24 h. Cells were starved with phosphate buffer solution (PBS) for 30 m, and the PBS was subsequently removed. Standards (rhEGF) and test samples were gradiently diluted 4 times in assay media after being pre-diluted to 10 IU/mL, and 200uL rhEGF serial dilutions were added to the cell plate prior to incubation at 37 °C with 5% CO_2_ for 48 h. MTT (20 uL) was added to each well and incubated at 37 °C with 5% CO_2_ for 4–6 h. A 10% SDS buffer solution (100 uL) was added into wells and incubated at 37 °C with 5% CO_2_ until color dots completely dissolved. Absorbance (OD_450_) values were determined using a SPECTRAmax plate reader (Molecular Devices, US). Resulting data were analyzed using Sigmaplot 11.0 (*see below*, “Data analyses”).

### NIH3T3 based assay (3T3)

Plates (96-well Costar) were seeded with 6000 NIH3T3 cells in RPMI-1640 with 3% FBS and incubated at 37 °C with 5% CO_2_ for 24 h. Cells were starved with RPMI-1640 with 0.4% FBS for another 24 h. Standards (rhEGF) and test samples were gradiently diluted 4 times in assay media after being pre-diluted to 50 IU/mL, and 200 uL rhEGF serial dilutions were added to the cell plate prior to incubation at 37 °C with 5% CO_2_ for 72 h. The steps below are the same as for CRI-3T3, include adding MTT, SDS, and reading.

### Bland-Altman analysis

Agreement between CRI-3T3 and 3T3 results was measured with a Bland-Altman plot. A total of 34 batches of rhEGF were measured with the two methods and the relative potency calculated. Each of the 34 samples was then represented on the graph by assigning the log-transformed average of two measurements as the abscissa (x-axis) value and the ratio of CRI-3T3 vs. 3T3 assay as the ordinate (y-axis) value.

### Data analyses

Data analysis consisted of statistical models used to calculate the EC_50_ value as well as statistical techniques for method validation. A four-parameter logistic model (Section 5.3 of European Pharmacopoeia) was used to calculate the relative potency, dose response, and linear range for NIH-3T3. Statistical techniques for method validation involved the coefficient of variation (CV) recovery rate. Analyses were carried out using Sigmaplot 11.0 for EC_50_ calculations and GraphPad Prism 7 for method validation. All data are presented as mean ± SEM, if not stated otherwise. Differences between groups were considered statistically significant at P < 0.05. Data presented is representative of two or more independent experiments, unless indicated otherwise.

## Materials

### Reagents

NIH3T3 and HEK293 were purchased from ATCC. RPMI 1640 (11835), DMEM(1929914) and fetal bovine serum (FBS) (10099) were purchased from Gibco, and Purinomycin (J593) was purchased from Amresco. Cell Counting Kit-8 (CCK-8) (HY-K0301) was purchased from MCE (MedChemExpress). GeCKO v2 2 vector system (lentiGuide-Puro) (1000000053) was purchased from Addgene.

### RhEGF and other cytokines

The National Standard for rhEGF (5800 IU/uL) is preserved by our department. RhEGF and other cytokines, including recombinant human erythropoietin (rhEPO), recombinant human granulocyte colony-stimulating factor (rhG-CSF), recombinant human granulocyte-macrophage colony-stimulating factor (rhGM-CSF), interleukin-2 (IL-2), interleukin-11 (IL-11), interferon γ (IFNγ), and interferon α-2b (IFNα-2b) were provided by different manufacturers.

## Data Availability

The data and materials described in the manuscript are available from the corresponding author on reasonable request.
